# Hybrid Microwave/Solar Energy Harvesting System Using 3D-Printed Metasurfaces

**DOI:** 10.3390/ma17235969

**Published:** 2024-12-05

**Authors:** Argyri Drymiskianaki, Zacharias Viskadourakis, George Kenanakis

**Affiliations:** 1 Department of Materials Science and Technology, University of Crete, GR-70013 Crete, Greece; 2 Institute of Electronic Structure and Laser (IESL), Foundation for Research and Technology—Hellas (FORTH), GR-70013 Crete, Greece; zach@iesl.forth.gr

**Keywords:** broadband energy harvesting, solar energy, microwave energy harvesting, metamaterials, 3D-printing, stereolithography

## Abstract

In this study, a hybrid energy harvesting system based on a conventional solar cell combined with 3D-printed metasurface units is studied. Millimeter-scale metasurface units were fabricated via the stereolithography technique, and then they were covered with conductive silver paint, in order to achieve high electric conductivity. The performance of single, as well as two-unit metasurface harvesters, was thoroughly investigated. It was found that both of them produced voltage, which peaks at their resonance frequency, demonstrating efficient energy harvesting behavior in the microwave regime. Then, the metasurface units were connected with a commercially available photovoltaic panel and the performance of the hybrid system was examined under different environmental conditions, modifying the light intensity (i.e., light, dark and shadow). It was shown that the proposed hybrid harvesting system produces a sizable voltage output, which persists, even in the case when one of the components does not contribute. Furthermore, the performance of the hybrid harvester is found to be adequate enough, although optimization of the harvesting circuit is required in order to achieve high efficiency levels. All in all, the presented experimental evidence clearly indicates the realization of a rather promising hybrid energy harvesting system, exploiting two distinct ambient energy sources, namely light and microwaves.

## 1. Introduction

Nowadays, contemporary rapid technological and industrial advancement has facilitated the need for ceaseless research centered on novel technologies and applications aiming at the utilization and administration of available energy resources. Sustainable methods of exploiting diverse forms of ambient energy (like solar radiation [1,2], mechanical [3,4] and acoustic vibrations [5,6], electromagnetic waves [7,8] and temperature gradients [9,10], to name a few), are increasingly becoming the main objective of modern research. Such methods can be categorized under the broad umbrella of energy harvesting (EH) technologies, where ambient energy is collected and converted into electrical power, making these systems of critical significance to the supply of low-power devices such as wireless sensor systems, flexible wearables, health monitoring devices, etc. [11,12,13]. Generally, such systems are supplied with periodically replaceable on-board batteries, making their use impractical, unsustainable and wasteful, due to the recurrent need for consumables [14]. Apart from that, unexpected battery discharge in cases where instant replacement is not feasible may cause severe issues in critical conditions. To address these issues, EH techniques can provide a partial, or even full, replenishment of the charging current to the energy storage medium of the device [15,16,17,18,19].

However, the efficiency of EH systems that rely on a specific kind of ambient energy can be heavily influenced by external conditions; for instance, the harvesting capability of a solar cell is rather high during the day, when solar illumination is strong, but it is totally suppressed during the night, when sunlight is absent. Even more, it can be severely reduced when weather conditions are not satisfactory (i.e., cloudy skies, shadows, etc.). In another typical example, harvesting devices, which rely on vibrations, could be very effective in busy buildings and streets with heavy traffic; however, their performance could be dramatically reduced during times when fewer people move into buildings, or fewer cars go around streets [20]. Considering the above-described examples, an EH system, which can harvest energy from more than one ambient source, could efficiently overcome such obstacles, continuously providing electrical power, regardless of the surrounding conditions. In other words, multiple energy harvesting technologies could be merged into a unified setup, comprising a so-called hybrid energy harvesting system (HEH) [21,22,23,24,25]. In particular, several discrete EH systems are electrically connected and are afterwards led to an energy combiner supplying specific storage elements. Through the above-described hybridization, the major conversion mechanism is selectively prioritized, enhancing the overall performance of the hybrid system [26]. Although an HEH system may involve more than two EH subsystems, for compatibility reasons recent research focuses on the coupling of two different EH technologies. Some state-of-the-art HEH cases involve the integration of mechanical/solar [27,28,29,30,31], solar/thermal [32,33,34,35] or even mechanical/thermal EH systems [36,37,38]. 

Moreover, nowadays, the abundance of microwave (MW) radiation emitted in free space from a plethora of communication systems such as mobile phones, Wi-Fi routers, Internet of Things (IoT) appliances, radars, and so on, enables the possibility of success-fully harvesting electromagnetic (EM) radiation in the millimeter-wave band. This emerging source of ambient energy enables the evolution of EH systems and devices performing in the MW frequency regime. MW EH systems are advantageous, since harvesting energy from microwaves is not affected by external conditions, such as light, traffic, movement, etc. Such an EH setup performs as long as MW signals are emitted. In this context, MW EH technologies could efficiently be combined with other sources such as mechanical, solar or thermal [25,39,40,41], for the development of a non-stop functional HEH setup.

So far, in the vast majority of cases, the MW harvesting component is represented by conventional antennas (either monopole or microstrip) [25,39,40,41,42,43,44,45,46,47,48,49]. This fact, however, is accompanied by limitations in terms of practicality, compatibility and cost [50]. On top of that, in order to successfully convert the harvested AC signal into DC output voltage, the inclusion of a rectifier is achieved via advanced simulation software, prior to proceeding to the fabrication stage [50,51].

Mitigating these challenges, extensive research centered on metamaterials and their energy harvesting properties has been conducted in recent years. Metamaterials comprise a class of artificial materials entailing precisely customized periodic structures capable of demonstrating EM phenomena in ways unachievable with natural materials [50,52]. The energy harvesting ability of metamaterials in the MW band has already been thoroughly studied, both theoretically and experimentally [53,54,55,56,57]. In contrast to conventional antennas, where EM radiation absorption may come at a severe expense of the overall performance (especially in telecommunication systems), metamaterial absorbers thwart signal attenuation effects due to the possibility of selectively tuning the desired operation bandwidth [58]. It is also known that efficient EM harvesters require the interstice between the unit cells forming the constructed metasurface (i.e., the planar array of symmetrically ordered meta-atoms) to be comparable to the operating wavelength [59]. Thus, the mm scale of the MW regime provides a convenient dimensionality regarding potential fabrication issues. Based on these assets, contemporary studies even attempt to combine conventional antennas with metamaterials, aiming to optimize the overall performance through their synergistic operation [60].

Even more, the utilization of additive manufacturing technology, in the development of metasurfaces, boosts their functionalization capabilities. Dedicated to the energy harvesting, 3D-printed harvesters, based on metasurfaces, have been successfully realized, and their performance is quite promising for their potential use in microwave harvesting [61,62]. Hence, a hybrid system, consisting of a solar panel and a 3D-printed MW harvester could be proposed. Such a system could produce electric power in a continuous manner, day and night, regardless of the light conditions. The amount of power produced from such an HEH system would be enough to support low-power devices. This capability opens up numerous potential applications with great environmental and economic impacts:

(a) Wearable electronics are one of the most promising areas for the application of HEH, since wearable devices can operate without the need for frequent battery replacements or recharging, which not only enhances user convenience, but also reduces electronic waste. Furthermore, wearables equipped with HEH systems can continuously monitor health metrics (e.g., heart rate, activity levels) without interruption due to low battery issues. This could improve healthcare outcomes by enabling real-time data collection. To continue, devices like smartwatches or fitness trackers can become more reliable and user-friendly when they do not depend on traditional power sources.
(b) The IoT ecosystem mainly relies on sensors that often need to be deployed in remote or hard-to-reach locations, where conventional power sources are impractical. First of all, sensors that harvest energy from the environment or human activities can operate independently, reducing maintenance costs associated with battery replacement. Secondly, IoT devices can be placed in a wider range of environments, including urban settings where human movement is prevalent, thus expanding the network’s reach and effectiveness. Last, but not least, continuous operation allows for more extensive data collection over time, leading to better analytics and insights in various applications such as smart cities and environmental monitoring.
(c) Remote sensing technologies benefit from HEH systems by providing sustainable power solutions for sensors used in various fields such as agriculture, wildlife monitoring, and disaster management. For example, integrated sensor nodes in civil infrastructure systems, such as water conservancy, power and communication facilities, roadways, railways, bridges, tunnels, buildings, agricultural facilities and environmental monitoring systems, are used to monitor and manage their functioning condition. Thus, harvesting systems, such as that proposed, could provide sufficient energy for all these devices, in a 24/7 way, overcoming the use of batteries and wire-base supplies. Moreover, these sensors can help track changes in ecosystems or monitor natural disasters, while minimizing the carbon footprint associated with traditional power sources, as reducing reliance on batteries lowers operational costs over time and minimizes logistical challenges related to energy supply in remote locations.
(d) The adoption of HEH systems has several positive environmental implications, since, by decreasing dependency on disposable batteries, HEH technology contributes to lower electronic-waste generation, while utilizing ambient energy sources reduces greenhouse gas emissions associated with manufacturing and disposing of batteries.
(e) From an economic perspective, integrating HEH systems into consumer products and industrial applications offers several advantages, since users save money on battery purchases over time as devices become self-sustaining, while companies investing in HEH technology may gain competitive advantages and access new markets focused on sustainability. Finally, through remote control, the monitoring and data acquisition can be accomplished automatically, without personnel needed to do so.

All in all, the HEH systems have transformative potential across various domains by enhancing device functionality while promoting sustainability. Their integration into wearable electronics, IoT devices, and remote sensing applications not only addresses practical challenges, but also contributes positively to environmental conservation efforts and economic efficiency. 

Up to now, several studies have explored the use of metasurfaces (MSs) as the MW component in an HEH system. In most of them, researchers primarily focus on the challenging and demanding process of designing and optimizing the contextual setup via specialized simulation methods. In the current study, we propose an HEH system consisting of a commercially available solar cell and 3D-printed MS units. Interestingly, to the best of our knowledge, there is not yet any experimental approach on MW/Solar HEH systems in which 3D-printed MSs are incorporated. In addition, the hereby-used MSs were fabricated employing the stereolithography (SLA) process. The use of 3D printing technology results in the growth of metamaterials and MSs with a complicated structure, in a quick, low-cost, eco/user-friendly manner [63,64]. Moreover, their specific geometry (cut-wire topology) has been extensively studied (theoretically and experimentally), regarding its EH properties, in the past [61,65], suggesting a novel MW harvester in the WiFi band. Therefore, the integration of such 3D-printed MSs, in an HEH system, sounds like an interesting idea. On the other hand, the experimental investigation of such a harvesting combination, under real-life conditions, will provide useful, more reliable information regarding its performance under realistic working conditions. 

As a first step, the EH capability of the 3D-printed meta-atoms was confirmed. Typical energy harvesting behavior was recorded, almost identical to that previously reported [61]. After that, an array of two cut-wire meta-atoms, connected in series, was also investigated, as a stand-alone EH device; such a device exhibited energy harvesting characteristics similar to that of the individual meta-atoms. However, the magnitude of the produced DC voltage was approximately equal to the sum of the DC voltages produced from each meta-atom separately. 

After comprehensive EM, as well as EH characterization of the 3D-printed meta-atoms, we constructed the HEH system by connecting the meta-atoms with a commercially available solar panel. The hybrid system exhibited good EH ability over a wide frequency range from 1 to 5 GHz. The stable DC output voltage of the solar cell was enhanced when the MS resonated. The efficiency of the HEH system was relatively low. The parameters affecting the low performance of the hybrid setup were thoroughly discussed. Furthermore, we studied the hybrid system with respect to luminosity. It was found that the produced voltage output was affected by luminosity; however, in dark conditions the HEH system still produced voltage output, due to the presence of the MSs. All in all, experimental results suggest that an HEH system, in which 3D-printed MSs are incorporated, is capable of selectively exploiting two distinct energy sources under different ambient conditions.

## 2. Materials and Methods

### 2.1. Meta-Atom Fabrication

**Figure 1:**
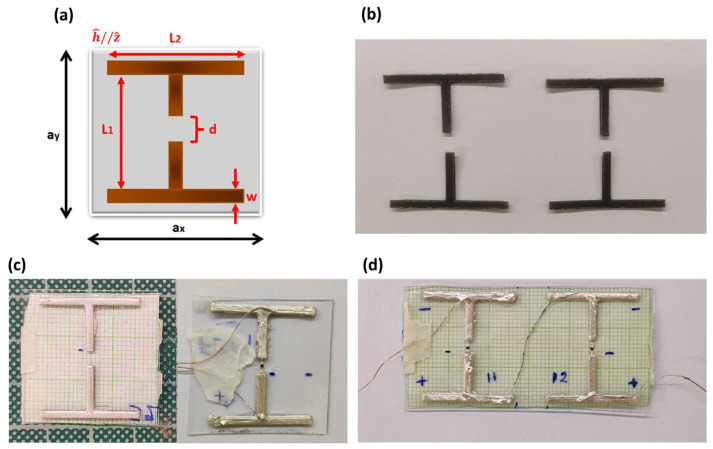
(**a**) Graphical representation of the cut-wire unit with the characteristic dimensions denoted. (**b**) Typical image of the 3D-printed structures before silver paint coating is applied. (**c**) Single-cell meta-atoms, before (**left**) and after (**right**). the insertion of a Schottky diode in the gap. (**d**) Two-unit array, with MS unit cells electrically connected in series.

MS units of cut-wire topology (Figure 1a) were fabricated utilizing stereolithography (SLA) 3D-printing technology. This geometry has been theoretically studied [65] for energy harvesting applications in the microwave regime. Through comprehensive full-wave numerical simulations, the meta-atom dimensions have been optimized, so that they exhibit a harvesting efficiency of 50% for incident power densities of WiFi signals. Corresponding experimental results for 3D-printed meta-atoms of similar dimensions show a moderate harvesting efficiency of ~7% [61]. Hence, the meta-atom geometry used here has been both theoretically and experimentally studied in the past. 

For the printing purposes, a commercially available SLA printer was used (Form 3+, Formlabs Inc., Somerville, MA, USA). Employing an ultraviolet laser (405 nm optical wavelength, 250 mW laser power and 85 µm spot size), the amount of photosensitive resin entailed in the tank is cured and subsequently solidified into a 3D object. The precise meta-atom design was made using Tinkercad software (2024 online version, Autodesk Inc., San Rafael, CA, USA), an open-source online platform intended for 3D modeling. The corresponding geometrical characteristics of the MS are shown in Table 1. After developing the appropriate drawing, it is transformed to CAD file, which is imported to the printing software (PreForm, Formlabs Inc., Somerville, MA, USA), signaling the printing initiation. Additional parameters relevant to the printing conditions concern the photopolymer type and the printing time, as well as the printed layer thickness. In our case, layer thickness was set at 0.02 mm, whereas the resin cartridge was supplied by the manufacturer (Draft V2 Resin, Formlabs Inc., Somerville, MA, USA). The printing area of the 3D printer allows for simultaneous growing of several meta-atoms, reducing both the printing time and printing consumables. Once the printing process is complete, the samples are ultrasonically cleaned using isopropanol.

After the construction of the meta-atoms, multiple thin layers of conductive silver paint were applied uniformly on the surface of each component. Silver paint exhibits high electrical conductivity, σ~10^5^ (Ω·cm)^−1^, turning the insulating polymer-based components into highly conductive elements. Next, the T-shaped structures were paired and glued on a transparent printing sheet of thickness t~1 mm and dimensions a_x_, a_y_ (Table 1). During the placement, the appointed gap value was ensured with the use of a removable piece of millimeter paper on the reverse side, as depicted in Figure 1c.

### 2.2. Electromagnetic Characterization of the Meta-Atom

The EM behavior of the constructed MS units was investigated through S-parameter measurements in the MW regime. In particular, all studied meta-atoms were measured using a combination of a P9372A Vector Network Analyzer (VNA) (Keysight, CA, USA) and a WR284 waveguide. Details regarding the setup and the measurement procedure were previously described [61]. All MSs were characterized in transverse electric (TE) mode, with the electric field component of the emitted EM wave being constantly perpendicular to the propagation direction, defining a zero angle of incidence between the electric field vector and the meta-atom gap (i.e., as in Supplementary Figure S1). Such a configuration has previously been proposed for cut-wire meta-atoms [61].

### 2.3. Electronic Circuit for Microwave-to-Dc Signal Conversion

In order to harvest MW radiation, the incident AC signal must be converted to DC output. The simplest way to do so is with the insertion of a diode into the meta-atom gap, as previously proposed [61]. More specifically, we used the HSMS 282B Schottky diodes (HSMS-282B-BLKG, Broadcom, CA, USA), due to their low voltage drop and fast switching times, which make them appropriate for applications in the GHz range [49]. Notably, other members of the HSMS 28x diode family have been used in rectification circuits of high-performance microwave harvesters [18,66,67,68]. It should be also noted that in the case of the MS array, it was crucial for the diodes to be inserted with the same polarity in each unit cell in order to obtain a total DC output signal. Although this type of signal rectification is far from optimal, we opted for it due to its simplicity and its already confirmed operability [61,62]. The design and the integration of an optimal rectifying circuit for this specific MS is a challenging issue to resolve. Thorough and complex computational methods are required, which are beyond the scope of the present study.

### 2.4. Energy Harvesting Properties of the Meta-Atom

In order to perform EH experiments of the meta-atoms, we employ the experimental setup proposed in [61]. The experimental setup consists of a signal generator (APSIN 6010HC, Ana Pico AG, Switzerland), transmitting in the 300 KHz–6 GHz frequency range, a double-ridged broadband horn antenna (BBHA 9120B, Schwarzbeck Mess-Elektronik oHG, Germany) and a high-precision digital multimeter (SDM3065X-SC, Siglent Technologies, Shenzhen, China) (as shown in the Results section). This specific type of horn antenna was chosen because of its wide operation bandwidth. The studied meta-atom is placed vertical to the cross-section of the antenna, while the free ends of the copper wires attached onto the meta-atom were connected to the multimeter measuring the DC voltage, across the diode. It is obvious that all measurements were performed in free-space, in contrast to other studies, where measurements were performed in an anechoic chamber [44,49,69]. The open-space measurements provide a more realistic approach concerning the performance of the EH system in real environmental conditions. However, as expected, free-space measurements suffer from unwanted diffusion, EM interference or reflection phenomena, which negatively affect the EH performance of the meta-atom. To reduce such parasitic effects, the distance between the horn antenna and the sample is kept as short as possible, in order to obtain adequate EM response, and consequently to enhance the signal-to-noise ratio. Notably, the afore-described experimental setup has already been successfully used for energy harvesting studies [61,62]. 

As discussed above, the meta-atom is placed in front of the antenna cross-section, maximizing the incident power absorption and therefore the DC output voltage transformed through the diode. The harvesting characterization of the meta-atom includes several experiments. At first, we measured the open-circuit DC voltage (V_OC_) as a function of frequency, under constant horn output power. In such experiments, we investigate whether or not DC voltage evolves at the frequency range where the meta-atom resonates. After that, V_OC_ as a function of horn output power, at resonance frequency, is measured in order to find out the power range where the meta-atom harvests energy without being saturated. Finally, by connecting several resistances parallel to the diode, the DC voltage output (V_OUT_) is measured at resonance frequency, for the determination of the maximum electric output power P_OUT_, for the estimation of the harvesting efficiency of the meta-atom. Obviously, similar experiments were performed for both the single-meta-atom and for the double-meta-atom array.

### 2.5. Solar Cell Characterization

The PV module used in this study has been purchased through the local market in Greece, and it consists of a commercially available amorphous Silicon solar panel with dimensions $\left(13\times 12.6\right)$ cm and total effective area $A_{eff}=163.8$ cm^2^. To confirm its manufacturer specs, the solar cell was characterized according to standard procedures, conducting current–voltage measurements for variable resistance-load values [70]. The corresponding experimental setup and experimental results are shown in Supplementary Figure S2 and Figure S3, respectively.

### 2.6. Hybrid EH Setup

**Figure 2:**
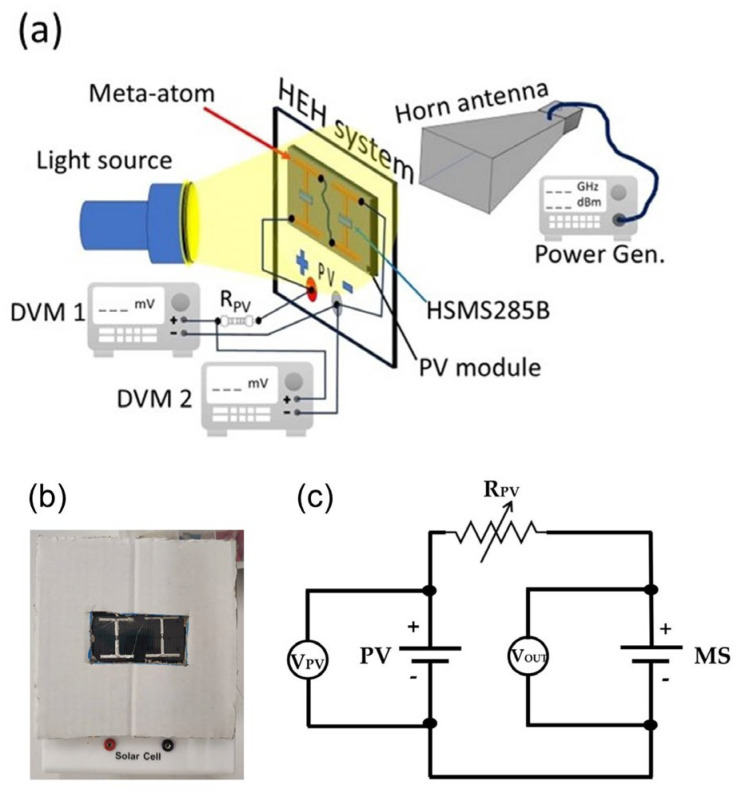
(**a**) Drawing of the experimental setup used to study the behavior of the HEH system. (**b**) Close–up view of the MS mounted on top of the masked solar cell. (**c**) The equivalent circuit diagram of the hybrid EH setup.

After confirming the efficient performance of both the 3D-printed MS unit and the 2-unit array, we proceeded to their electrical connection with the solar panel, for the formation and characterization of the HEH system. The corresponding experimental setup and circuit is presented in Figure 2a. More specifically, the MW component is directly attached to the solar panel and connected with it, through Cu leads, as previously proposed [39,43,71,72]. Due to its large area, compared with the meta-atom, the solar panel has been masked (Figure 2b). In such a way, the solar panel and the MS exhibit similar active areas, which is crucial for a direct comparison of their harvesting performance. A variable-resistance R_PV_ was inserted in series with the PV component, in order to regulate its voltage overshoots, preventing the diode from being damaged. The harvested DC output voltage was measured through a digital voltmeter (SDM3065X-SC, Siglent Technologies, Shenzhen, China—DVM2 in Figure 2a) connected parallel to the diode’s terminals. A second multimeter (SDM3055-SC, Siglent Technologies, Shenzhen, China—DVM1 in Figure 2a) was used to track the output of the solar cell. The analogous electronic circuit is presented in Figure 2c. The HEH system was put opposite to a light source in a distance of ~30 cm, while a horn antenna was placed just behind the HEH system, as shown in Figure 2a. Such an arrangement fulfills several issues that must be taken into consideration. First of all, the use of a small number of meta-atoms in the constructed MSs requires minimizing the distance from the horn antenna. On the other hand, this limitation did not apply to the solar cell when examined individually; the harvested output voltage was found to be almost stable over the distance of the light source (in the scale of centimeters). Due to this fact, the most convenient method for the HEH system was for it to be placed in front of the cross-section of the horn antenna (Figure 2a). During experiments, the whole setup was covered with opaque blackout curtains in order to block any background light that could act cumulatively on the PV component. 

Such a connection of both PV and meta-atom modules enables the capability of the HEH to harvest energy, even in cases when one of the counterparts does not contribute. For example, in the dark, the PV does not produce any power, while the meta-atom will harvest energy. In this way, the HEH system is certain to produce a constant output voltage, regardless of the light conditions.

This assembly has been tested under various conditions, in order to accurately evaluate its performance. In particular, the system’s response as a function of the R_PV_ load, incident light percentage and effective area of the PV module has been examined. Within this framework, the following states of operation have been tested, alternating sequentially the type of the available ambient energy source. Three main states were examined: (i) ‘ON STATE’ (when both light and MW radiation are available, (ii) ‘MW ON + PV OFF STATE’, when only the horn antenna emits (MW energy source), and (iii) ‘MW OFF + PV ON STATE’, when the only ambient energy is emitted from the light source. First, a -atom was utilized as the MW component in the hybrid system and then the 1 × 2-meta-atom array was been tested.

## 3. Results and Discussion

### 3.1. EM Properties of the MW Component

**Figure 3:**
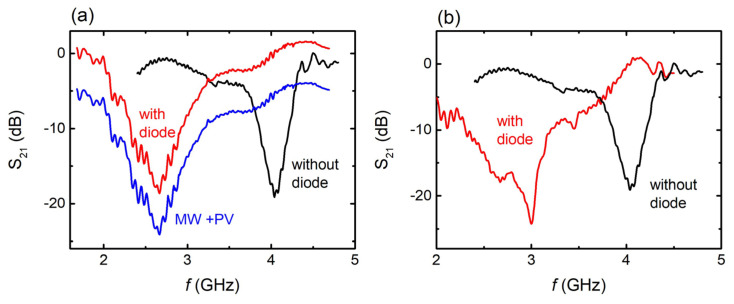
(**a**) S_21_ vs. frequency of single meta-atom with (red solid line) and without (black solid line) diode. Corresponding S_21_ vs. *f* spectrum, for hybrid system. (**b**) S_21_ vs. frequency for two–meta–atom array, before (black solid line), and after (red solid line) the insertion of the Schottky diode in the meta-atom gap.

The EM response of the 3D-printed meta-atom is investigated and S_21_ vs. frequency spectra are shown in Figure 3. 

In particular, in Figure 3a, S_21_ vs. frequency spectra are shown for the single meta-atom, with (red line) and without (black line) the diode inserted into the gap. In the absence of the diode, the meta-atom exhibits a strong resonance at ~4 GHz. The obtained resonant response is indicative of absorption of the incident MW energy (corresponding to reflection—the S_11_ vs. frequency spectrum does not show any feature in the whole measured frequency range). By incorporating the diode into the gap of the MW meta-atom, a resonance shift is observed, towards lower frequencies, i.e., at ~2.6 GHz. Such an electromagnetic behavior is in perfect agreement with previous studies obtained for fused filament fabricated meta-atoms [61]. Furthermore, the EM response of the meta-atom array after the intervention of the solar cell in the hybrid setup is presented (solid blue line). A downward shift of ~5 dB is uniformly observed on the measured S_21_ magnitude across the whole frequency range. This intensity reduction is attributed to the presence of the PV module between the MW source and the meta-atoms. However, after the inclusion of the solar cell, the strong resonance peak around ~2.6 GHz is still visible, indicating that the EM response of the meta-atom is hardly affected by the presence of the PV component. Moreover, the EM behavior of the 2 × 1 MW array is shown in Figure 3b. The array resonates at ~4 GHz, while the connection of the diodes results in a frequency shift, and thus the resonance frequency goes to ~3 GHz. This behavior is qualitatively similar to the behavior of the single meta-atom.

In both cases, the conspicuous resonant shift is attributed to impedance mismatch between the MS and the diode [73]. As already discussed in Section 2.3, the diode is inserted into the gap of the MS, as a rectifier. Conventional high-frequency rectifiers are complicated electronic circuits which can effectively perform at a specific frequency regime, depending on factors such as the electronic components used. As long as the frequency range where the rectifier shows maximum performance matches the resonance frequency regime of the MS, then it is considered that the impedance match is achieved and the rectification efficiency is maximized. Otherwise, impedance mismatch would have serious effect on the rectification process, leading to the suppression of the overall MS harvesting behavior. Thus, optimization of the rectification circuit, in order to perform in a similar frequency range with the MS, becomes vital for an efficient microwave MS harvester. However, it is a challenging electrical engineering issue, since extensive computational simulations are required, and complicated electrical simulators must be employed. In the current study, the main goal is to explore the prospect of integrating a 3D-printed MS into a hybrid energy harvesting system. Therefore, the investigation into optimizing the impedance mismatch could be the main aim of future work.

Finally, a slight broadening of the resonance peaks can be observed both in Figure 3a, and b. This outcome can be attributed to the presence of the diode, and enables the possibility of the MS operating in a wider frequency range [74,75,76]. Therefore, this fact can be proven advantageous, as it can be exploited to widen the operation bandwidth of the MW component.

### 3.2. Energy Harvesting Properties of the Metasurfaces 

**Figure 4:**
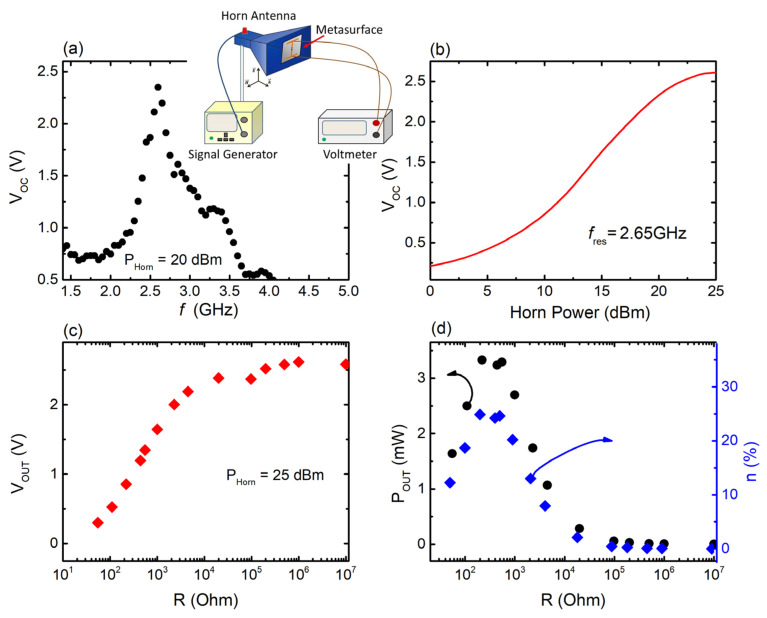
(**a**) Open-circuit voltage (V_OC_) vs. frequency for single MS unit. Graphical illustration of the experimental setup used to measure the harvesting performance of the MS is shown in the inset. (**b**) V_OC_ as a function of the horn antenna output power. (**c**) The dependence of output voltage (V_OUT_) on the resistance load, at resonance frequency. (**d**) Output power (black points) and calculated efficiency (blue points) as a function of resistance load for the single MS unit.

Figure 4a depicts the open-circuit voltage V_OC_ delivered by the 3D-printed meta-atom (as defined in Section 2.2), with respect to the frequency. It can be clearly seen that the V_OC_ response of the single MS unit demonstrates a strong maximum at ~2.65 GHz, with peak value V_OC, max_~2.4 V. The frequency where the V_OC_ maximum is located coincides with the resonance frequency of the corresponding S_21_ minimum (Figure 3a), identifying this output voltage as an EH outcome. Notably, such observed behavior is in agreement with previous studies concerning 3D-printed cut-wire MSs [61]. No other noteworthy maxima attributable to resonance phenomena were noted; any other detected V_OC_ fluctuations are possibly attributed to the intrinsic non-linear profile of the diode [62,72,77].

The open-circuit response of the single-meta-atom MS as a function of the horn antenna power is presented in Figure 4b. As can be seen, V_OC_ values follow an increase in the output power, confirming that the measured voltage originates from the rectified harvested signal. For maximum horn power (25 dBm), we obtain Voc~3.1 V, at resonance frequency. However, for horn power greater than ~23 dBm, the system enters a stage where Voc seems to saturate. It means that the system reaches its harvesting limit; thus, increments of the horn power above ~23 dBm would not result in any notable increase in the V_OC_, regardless of the incident power increment. Consequently, the harvesting efficiency of the device would also be depressed. For this reason, the signal generator output power was kept at a fixed value of 20 dBm, just before MS enters the saturation regime. 

Figure 4c depicts the output voltage V_OUT_ of the MSs with respect to the resistance load R. As can be seen, V_OUT_ increases with increasing R, whereas for R > 100 kOhm a voltage plateau is reached, exhibiting values comparable to V_OC_. Figure 4d describes the output power produced by the harvester, as calculated through the formula $P_{OUT}=V_{OUT}^{2}/R$ (black solid circles). As can be seen, the meta-atom exhibits maximum P_OUT_ for l R_opt_ ~ 550 Ω. The observed behavior is almost identical to the behavior observed for other MSs of the same topology [61]. Moreover, the efficiency of the harvester is also presented in Figure 4d (blue solid rhombs), using the relation $\eta \%={(P}_{OUT}/P_{IN})\times 100$ [61,62]. Τhe 3D-printed meta-atom demonstrates a maximum efficiency of 24.6%, as depicted in Figure 4d. The estimated efficiency is significantly greater in comparison with previous studies, where for FDM-printed MSs a peak efficiency of ~8% is obtained [61]. This may be attributed to the different type of diodes used in the current study. Analogous enhancement of the harvesting efficiency has been observed for traditionally PCB-grown cut-wire meta-atoms (Supplementary Figure S4), when combined with the diodes used here. In general, electrical characteristics of the diodes could have a direct impact on the overall effectiveness of signal rectification. Apparently, the specific type of diode used in this study seems to be compatible with the electrical characteristics of the cut-wire geometry, leading to an increase in the MS efficiency.

**Figure 5:**
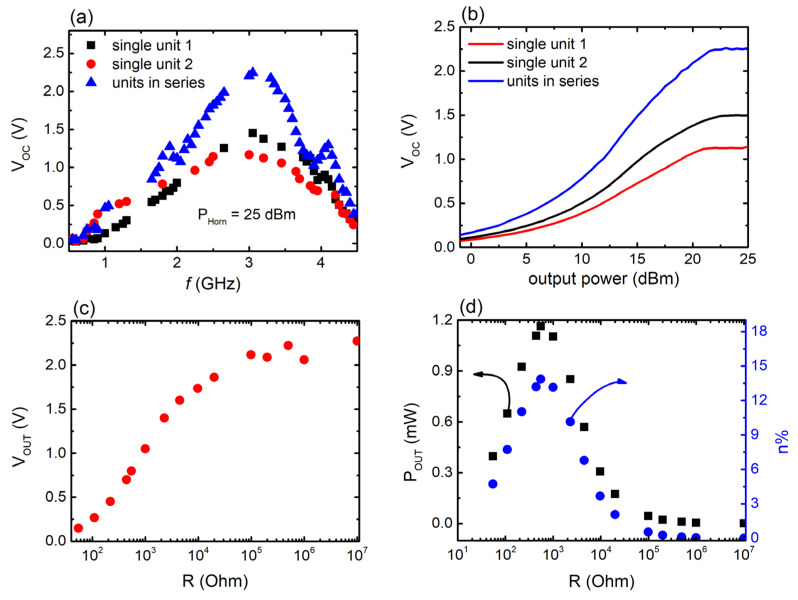
(**a**) Open-circuit voltage (V_OC_) with respect to frequency for the 3D-printed meta-atom array. (**b**) V_OC_ as a function of the signal generator power for electrically connected meta-atoms; the contribution of each unit cell is separately measured. (**c**) The dependence of output voltage (V_OUT_) of the array on the resistance load, at resonance. (**d**) Output power (black squares) and calculated efficiency (blue points) for the 3D-printed meta-atoms array.

Τhe EH response of the 1 × 2 MS array is demonstrated in Figure 5.

As can be seen from Figure 5a, the array exhibits a broad, but well-defined voltage peak at ~3 GHz (Voc~2.3 V), which perfectly coincides with its resonance frequency (Figure 3b), suggesting energy harvesting. The fluctuations observed beyond resonance region are mainly attributed to the nonlinearity of the diodes [62,72,77]. On top of that, as depicted in Figure 5a, one can discern that, at its peak, the V_OC_ of the array is almost equivalent to the sum of the peak voltages derived from the individual unit cells when in resonance, corroborating the serial electrical connection, although in the non-resonant regime, this is not the case. The cumulative nature of the series connection is also seen in Figure 5b, which describes the dependence of the measured V_OC_, on the horn output power. Moreover, it is shown that, for intensities greater than 20 dBm, V_OC_ saturates, similarly to the single-meta-atom behavior (i.e., as shown Figure 4b). At this point, it must be noted that the V_OC_ of the array appears to be less than the V_OC_ measured for single meta-atom, as presented in Figure 4a. Furthermore, the V_OC_ values of each meta-atom of the array are almost half of the V_OC_ magnitude, as shown in Figure 4a. Such a discrepancy could be the result of several factors: first of all, the presence of copper leads, used for the electrical connections of the unit cells (Figure 1c,d), has most likely induced a non-negligible amount of noise, which in turn leads to a significant decrease in the individual peak voltages, suppressing the overall output and introducing fluctuations to the measured signal. In addition, the position of the array in front of the horn antenna suggests the off-center location of each single meta-atom, with respect to the antenna cross section. Considering the non-plane profile of the incident wave coming out of the horn [78], it could lead to a suppressed wave energy absorption and a resultant low Voc. In the case of the single meta-atom, which is exactly centered with respect to the antenna cross section, the incident wave energy absorbed by the meta-atom is maximum, resulting in a maximum V_OC_ magnitude. Even though all the above-discussed contributions could cumulatively suppress the overall V_OC_, the phenomenon still survives, and it is sizable, indicating the effectiveness of the MS array as an energy harvester in the microwave regime.

Figure 5c describes the output voltage V_OUT_ of the array with respect to the resistance load R. As in the case of the single meta-atom, the obtained V_OUT_ increases with increasing R, whereas for loads greater than 10 kOhms a V_OUT_ plateau is observed, coinciding with the V_OC_ magnitude. Moreover, in Figure 5d, the output power P_OUT_ and corresponding efficiency n% are presented for the MS array. Both maximum P_OUT_ and n% values are obtained for R_opt_~500 Ohm, similarly to the single meta-atom. For such a load, the P_OUT_~1.2 mW and n%~14% are the values. Apparently, both P_OUT_ and n% are reduced, compared to the single-cell harvester (Figure 4d). The reduced efficiency obtained for the MS array, could be attributed to the factors previously discussed, which lead to the suppressed V_OC_ values. 

### 3.3. Hybrid MW/PV System Performance

**Figure 6:**
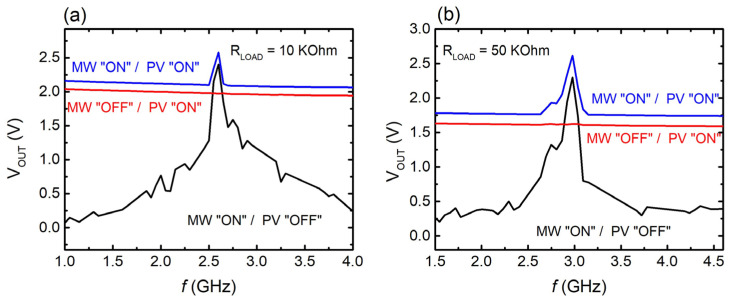
Output voltage V_OUT_ of the hybrid EH system as a function of frequency for (**a**) single unit-cell and (**b**) meta-atom array.

The behavior of the HEH system at different operational states is presented below (Figure 6). 

More specifically, Figure 6a shows the V_OUT_ vs. frequency, regarding the setup, including the PV and the single meta-atom. It is clearly seen that when only the PV component harvests (PV, “ON” state, MW “OFF” state), the harvested voltage was derived from the PV module and remained constant at ~2 V across the whole spectral region. On the other hand, when only the MW component harvests energy (PV “OFF”, MW “ON”), a voltage peak is observed at 2.6 GHz, where the meta-atom resonates, consistent with previous discussed data. Thus, it is proven that in the proposed HEH system, harvesting counterparts can contribute in complement with each other, producing electric power in a continuous mode. Furthermore, when both components are in the “ON” state, an increase in the output voltage around the resonance region is traced (blue line). This behavior is in qualitative agreement with previous studies regarding similar HEH systems [43,72,79,80]. Interestingly, in this frequency regime, total voltage output equals the voltage output of the MW component; i.e., a peak voltage of ~2.6 V is obtained. For frequencies away from resonance, the total voltage output equals that coming from the PV component. Therefore, when in resonance, the MW component seems to mainly contribute, while it is the opposite case for the non-resonant frequencies. Nevertheless, regardless of the frequency range, a constant voltage output is recorded, enabling the capability of the proposed harvesting system to continuously produce energy, using more than one ambient energy source. Here, it must be stressed that the resistance load R_PV_ = 10 KOhm did not impede, to a great extent, the current produced by the solar cell. However, its use is vital for the protection of the diode; using a low R_PV_ load results in large PV currents, which would cause diode failure. On the other hand, further increase in the R_PV_ substantially suppressed the total output voltage of the hybrid system.

Correspondingly, Figure 6b shows the voltage output in the HEH system, in which the two-meta-atom array is included. The general behavior of the system is identical to that described previously. Here, a voltage peak V_OUT_ of ~2.5 V is derived at ~3 GHz, when both PV and MW components harvest energy. Thus, it is shown that the proposed HEH system can be upgraded when using MS arrays instead of single-meta-atom units. 

The overall performance of both the HEH systems can be defined through the calculation of their efficiencies. Table 2 summarizes some characteristic quantities for the EH components, as well as the calculated efficiencies. Notably, the efficiency of the hybrid system was calculated using the formula $\eta_{combining}\%=\left[P_{OUT}^{hybrid}/\left(P_{MW}^{IN}+P_{PV}^{IN}\right)\right]\times 100$, as previously proposed [41], where $P_{OUT}^{hybrid}$ is the output power of the HEH system, achieved with the optimal resistance load, $P_{MW}^{IN}$ is the output power of the MW component for the optimal resistance load, and $P_{PV}^{IN}$, is the output power of the PV, achieved in optimal resistance load. As can be seen, the efficiency of the hybrid system is significantly lower compared to that of the individual components. This is mainly attributed to the already low efficiencies of the contributing harvesters. Moreover, potential impedance mismatches between the electrical components could also contribute to the low efficiency; optimization of the synergetic operation of the individual energy harvesters, minimizing losses of the system [81,82], is obviously required, in order to enhance the performance of the system. Another thing that should be taken into consideration is that the efficiency values of the hybrid system have been calculated with respect to the unitary cell of the individual components, ensuring their equal contribution; within this framework, it is reasonable that the calculated efficiency would be lower, compared with similar hybrid RF/solar EH systems [43,72,79]. Regardless of its sufficiently low performance, experimental evidence clearly shows that the proposed PV/MW setup could be a promising candidate for HEH applications.

**Figure 7:**
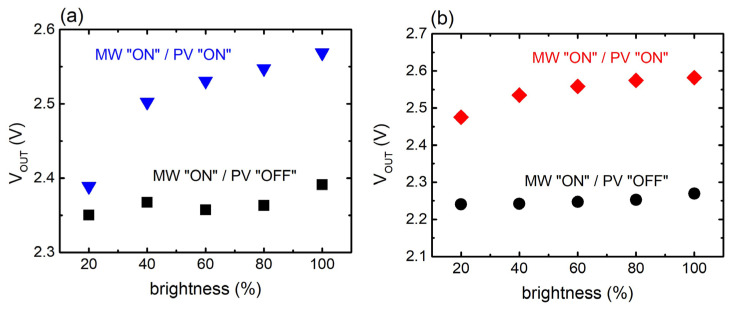
Output voltage V_OUT_ of the hybrid EH system as a function of ambient light brightness for (**a**) single-meta-atom system and (**b**) meta-atom array.

Furthermore, the behavior of the hybrid system with respect to different lightning conditions is investigated (Figure 7). Particularly, the behavior of the single-unit/PV system, is presented in Figure 7a. When in resonance, and with the PV component in the “OFF” state, the voltage output remains constant, regardless of the brightness of the light emitted. Upon the activation of the PV component (“ON” state), the voltage output increases with increasing brightness of the light. A similar response is also obtained for the two-MS unit/PV system (Figure 7b). Therefore, it is clearly shown that the proposed HEH system is affected by the light conditions. 

## 4. Conclusions

In this study, a hybrid EH system employing a 3D-printed metamaterial-based MW energy harvester and a solar cell was examined. The MW harvester was of cut-wire geometry, and it was fabricated via the SLA 3D-printing process. Both the single-meta-atom as well as the two-meta-atom arrays were examined. Both MSs were proven capable of harvesting ambient MW radiation, demonstrating strong resonance peaks around 2.6 GHz and 3 GHz, respectively, a region typically observed in WiFi signals and other 5G communication technologies. The performance of the single-meta-atom harvesters was found to be greater than that of the other 3D-printed harvesters previously reported, which was attributed to the diode used for the rectification process. On the other hand, the performance of the MS array was found to be lower than expected, possibly due to unwanted coupling and fabrication limitations, as previously discussed. However, the obtained output voltage values are quite promising for EH applications.

The merging of 3D-printed EH meta-atom with a PV module into a unified system has enabled the possibility of exploiting a different ambient energy source, depending on the availability, addressing the hitherto significant obstacle of photovoltaic performance instability. The performance of the hybrid system was tested under different operational states. The presented results show that when only MW radiation is available, the DC output of the system is derived solely from the MS, while in cases where both ambient sources are available, the system is almost totally contributed to from the MW component when in resonance, while it is fully supported from the PV across the rest of the spectral region. Qualitatively similar behavior was also observed for the MW array/PV system, as well. Such behavior can be plausibly explained, considering the corresponding electronic circuit, in both cases. Additionally, the output of the hybrid system was found to be stable under brightness fluctuations, due to the MS inclusion. Hence, the proposed hybrid setup was proven to be capable of providing an uninterrupted power output in the range of ~mW, enabling the opportunity for seamless energy harvesting on a constant basis.

## 5. Future Work

In the present study, the proposed HEH system is proven to be an affective candidate for limitless energy harvesting. Nevertheless, several challenging issues came up, which could be subjected to further investigation in the future. Most of all, the improvement in its harvesting efficiency becomes vital. In this context, future studies could be accordingly directed toward this. More specifically, the enhancement of the MW component harvesting efficiency requires the optimization of the rectification circuit. Thus, the development of a rectifier suitable for the studied cut-wire topology, leading to the boosting of the MS’s harvesting capability, sounds like the number one mission to accomplish. In addition, other topologies could also be tested, in combination with PV modules. Recently, an engraved MS of toroidal geometry has been explored, which exhibited rather promising harvesting behavior at ~5 GHz, enabling its use in harvesting in 4G/5G bands [62]. In addition, the incorporation of power management electronics would further improve the harvesting capability of the proposed HEH system. Considering the above, it becomes apparent that appropriate research efforts can be put towards the improvement of the performance of the proposed hybrid harvester.

Another interesting research issue is the feasibility of the upscale of the proposed HEH system, for use in urban environments and/or in industry. Therefore, a sizable expansion of the proposed HEH system is required. Apparently, large-area PV panels are already commercially available, and hence the key question is whether large areas of MSs can be constructed or not. Taking into account the existing advances in additive manufacturing, 3D-printing technology could be employed in the construction of such large panels. For example, each of those panels would be printed in complementary pieces, just like a puzzle, and then the pieces could be attached to each other, formatting a large MS panel. By appropriate optimization of the dimensions, the EM performance of such a panel can be adjusted for maximum power absorption and, consequently, for optimal harvesting performance. The existing technology could support such an upscale, while the construction time needed is directly proportional to the panel dimensions. The larger the pane, the more time required for its development. Moreover, other techniques could be proposed for MS deposition in large panels. In this context, in a recent study [83], the MSs of the cut-wire topology were grown in plasterboards and on wooden surfaces, for potential electromagnetic applications in large buildings. In conclusion, the construction of large panels of MSs seems to be achievable, suggesting a promising research direction, which will be followed up. 

In scaling up the HEH system, in order for it to be used for large-area applications, there are a couple of other challenging issues suitable for investigation. First of all, the conversion of the microwave signal, coming from such large MS panels, to a dc power, becomes puzzling. The rectification of the incident power coming from m^2^ scale panels of MSs probably preconditions the employment of high-power electronic circuits and power management devices performing in the GHz regime. Although such technology may exist, there is not any published investigation dedicated to it, to the best of our knowledge. Therefore, such studies would be of great interest. On the other hand, the use of such HEH systems in urban environments suggest their outdoor installation. Thus, the behavior of such system in extreme weather conditions (high/low temperatures, humidity, wind, etc.) is under discussion. The efficiency of photovoltaics under unconventional weather conditions has been thoroughly studied [84,85,86]. Additionally, the EM behavior of MSs in a humid surrounding varies [87,88,89], which will possibly result in a reduction of its harvesting capabilities. Consequently, appropriate investigation is required in order to achieve an HEH system capable of effective harvesting, regardless of the weather condition changes. 

Considering the above discussion, it becomes more than obvious that several research directions come up, through the current investigation, within the realization of a high-performance PV/MS hybrid harvester suitable for low-power applications. 

## Figures and Tables

**Table 1:** Dimensional characteristics of the constructed cut-wire units. Parameter h corresponds to the thickness of the meta-atom.

Parameter	Size (±0.1 mm)
L_1_	23.1
L_2_	22.8
a_x_, a_y_	30.0
g	3.0
h	1.0
w	1.6

**Table 2:** Output power P_OUT_, optimal resistance load and determined efficiencies for the single meta-atom, for two-meta-atom array, for PV, and for the HEH system. Quantities for the PV component are extracted from Supplementary Figure S3.

Ambient Source	Component	P_OUT_ (mW) (at f_resonance_)	R_opt_ (Ohm)	Efficiency η%
ΜW	single meta-atom	3.3	550	24.6
	meta-atom array	1.2	550	13.9
Solar	PV module	16	442	~20
MW/PV	Hybrid system	~0.67 ~0.14	9.77 k 50 k	3.5 (single meta-atom) 0.7 (meta-atom array)

## Data Availability

The raw data supporting the conclusions of this article will be made available by the authors on request.

## Supplementary Materials

The following supporting information can be downloaded at: https://www.mdpi.com/article/10.3390/ma17235969/s1, Figure S1: Orientation of the meta-atom into the waveguide, during its electromagnetic behavior study. Figure S2: (a) Schematic representation of the experimental setup used to derive the I-V characteristics of the solar cell. (b) The amorphous Si solar cell used in the HEH system. (c) The equivalent circuit diagram of Figure S2a. Figure S3: (a) I-V characteristics of the PV module at different lighting conditions. The effective area of the solar cell was reduced to match the affective area of the MS. (b) Output power over resistance load at various brightness levels. Figure S4: (a) Open-circuit voltage (V_OC_) vs. frequency for PCB-grown, single metasurface unit. (b) V_OC_ as a function of the horn antenna output power. (c) The dependence of output voltage (V_OUT_) on the resistance load, at resonance frequency. (d) Output power (black points) and calculated efficiency (red points) as a function of resistance load for the single metasurface unit.

## References

[1] Abdin Z., Alim M.A., Saidur R., Islam M.R., Rashmi W., Mekhilef S., Wadi A. (2013). Solar Energy Harvesting with the Application of Nanotechnology. Renew. Sustain. Energy Rev..

[2] Lau D., Song N., Hall C., Jiang Y., Lim S., Perez-Wurfl I., Ouyang Z., Lennon A. (2019). Hybrid Solar Energy Harvesting and Storage Devices: The Promises and Challenges. Mater. Today Energy.

[3] Zuo L., Tang X. (2013). Large-Scale Vibration Energy Harvesting. J. Intell. Mater. Syst. Struct..

[4] Wei C., Jing X. (2017). A Comprehensive Review on Vibration Energy Harvesting: Modelling and Realization. Renew. Sustain. Energy Rev..

[5] Horowitz S.B., Sheplak M., Cattafesta L.N., Nishida T. (2006). A MEMS Acoustic Energy Harvester. J. Micromechanics Microeng..

[6] Khan F.U. (2015). State of the Art in Acoustic Energy Harvesting. J. Micromechanics Microeng..

[7] Hwang J.H., Hyoung C.H., Park K.H., Kim Y.T. (2013). Energy Harvesting from Ambient Electromagnetic Wave Using Human Body as Antenna. Electron. Lett..

[8] Erkmen F., Almoneef T.S., Ramahi O.M. (2017). Electromagnetic Energy Harvesting Using Full-Wave Rectification. IEEE Trans. Microw. Theory Tech..

[9] Zabek D., Morini F. (2019). Solid State Generators and Energy Harvesters for Waste Heat Recovery and Thermal Energy Harvesting. Therm. Sci. Eng. Prog..

[10] Kishore R.A., Priya S. (2018). A Review on Low-Grade Thermal Energy Harvesting: Materials, Methods and Devices. Materials.

[11] Vullers R.J.M., van Schaijk R., Doms I., Van Hoof C., Mertens R. (2009). Micropower Energy Harvesting. Solid State. Electron..

[12] Beeby S.P., Tudor M.J., White N.M. (2006). Energy Harvesting Vibration Sources for Microsystems Applications. Meas. Sci. Technol..

[13] Harb A. (2011). Energy Harvesting: State-of-the-Art. Renew. Energy.

[14] Edwards R., Gould C. Review on Micro-Energy Harvesting Technologies. Proceedings of the 2016 51st International Universities Power Engineering Conference, UPEC 2016.

[15] Robinson B.H. (2009). E-Waste: An Assessment of Global Production and Environmental Impacts. Sci. Total Environ..

[16] Wang X., Gaustad G., Babbitt C.W., Bailey C., Ganter M.J., Landi B.J. (2014). Economic and Environmental Characterization of an Evolving Li-Ion Battery Waste Stream. J. Environ. Manag..

[17] Notter D.A., Gauch M., Widmer R., Wäger P., Stamp A., Zah R., Althaus H.J. (2010). Contribution of Li-Ion Batteries to the Environmental Impact of Electric Vehicles. Environ. Sci. Technol..

[18] Mousa Ali E., Yahaya N.Z., Nallagownden P., Zakariya M.A. (2017). A Novel Rectifying Circuit for Microwave Power Harvesting System. Int. J. RF Microw. Comput. Eng..

[19] Perna G., Bonacci F., Caponi S., Clementi G., Di Michele A., Gammaitoni L., Mattarelli M., Neri I., Puglia D., Cottone F. (2023). 3D-Printed Piezoelectret Based on Foamed Polylactic Acid for Energy-Harvesting and Sensing Applications. Nanomaterials.

[20] Du R., Xiao J., Chang S., Zhao L., Wei K., Zhang W., Zou H. (2023). Mechanical Energy Harvesting in Traffic Environment and Its Application in Smart Transportation. J. Phys. D Appl. Phys..

[21] Weddell A.S., Magno M., Merrett G.V., Brunelli D., Al-Hashimi B.M., Benini L. A Survey of Multi-Source Energy Harvesting Systems. Proceedings of the 2013 Design, Automation & Test in Europe Conference & Exhibition (DATE).

[22] Khaligh A., Zeng P., Zheng C. (2010). Kinetic Energy Harvesting Using Piezoelectric and Electromagnetic Technologiesstate of the Art. IEEE Trans. Ind. Electron..

[23] Guo L., Gu X., Chu P., Hemour S., Wu K. (2020). Collaboratively Harvesting Ambient Radiofrequency and Thermal Energy. IEEE Trans. Ind. Electron..

[24] Lorenz C.H.P., Hemour S., Liu W., Badel A., Formosa F., Wu K. (2015). Hybrid Power Harvesting for Increased Power Conversion Efficiency. IEEE Microw. Wirel. Components Lett..

[25] Gu X., Liu W., Guo L., Hemour S., Formosa F., Badel A., Wu K. (2018). Hybridization of Integrated Microwave and Mechanical Power Harvester. IEEE Access.

[26] Ryu H., Yoon H., Kim S. (2019). Hybrid Energy Harvesters: Toward Sustainable Energy Harvesting. Adv. Mater..

[27] Ahmed N.A., Miyatake M., Al-Othman A.K. (2008). Power Fluctuations Suppression of Stand-Alone Hybrid Generation Combining Solar Photovoltaic/Wind Turbine and Fuel Cell Systems. Energy Convers. Manag..

[28] Schlichting A., Tiwari R., Garcia E. (2012). Passive Multi-Source Energy Harvesting Schemes. J. Intell. Mater. Syst. Struct..

[29] Zheng L., Cheng G., Chen J., Lin L., Wang J., Liu Y., Li H., Wang Z.L. (2015). A Hybridized Power Panel to Simultaneously Generate Electricity from Sunlight, Raindrops, and Wind around the Clock. Adv. Energy Mater..

[30] Wang S., Wang X., Wang Z.L., Yang Y. (2016). Efficient Scavenging of Solar and Wind Energies in a Smart City. ACS Nano.

[31] Xu C., Wang Z.L. (2011). Compact Hybrid Cell Based on a Convoluted Nanowire Structure for Harvesting Solar and Mechanical Energy. Adv. Mater..

[32] Kraemer D., Hu L., Muto A., Chen X., Chen G., Chiesa M. (2008). Photovoltaic-Thermoelectric Hybrid Systems: A General Optimization Methodology. Appl. Phys. Lett..

[33] Park K.-T., Shin S.-M., Tazebay A.S., Um H.-D., Jung J.-Y., Jee S.-W., Oh M.-W., Park S.-D., Yoo B., Yu C. (2013). Lossless Hybridization between Photovoltaic and Thermoelectric Devices. Sci. Rep..

[34] Wang N., Han L., He H., Park N.H., Koumoto K. (2011). A Novel High-Performance Photovoltaic-Thermoelectric Hybrid Device. Energy Environ. Sci..

[35] Yang D., Yin H. (2011). Energy Conversion Efficiency of a Novel Hybrid Solar System for Photovoltaic, Thermoelectric, and Heat Utilization. IEEE Trans. Energy Convers..

[36] Kim S., Hyeon D.Y., Ham S.S., Youn J., Lee H.S., Yi S., Kim K.T., Park K.-I. (2021). Synergetic Enhancement of the Energy Harvesting Performance in Flexible Hybrid Generator Driven by Human Body Using Thermoelectric and Piezoelectric Combine Effects. Appl. Surf. Sci..

[37] Ko Y.J., Kim D.Y., Won S.S., Ahn C.W., Kim I.W., Kingon A.I., Kim S.-H., Ko J.-H., Jung J.H. (2016). Flexible Pb(Zr0.52Ti0.48)O3 Films for a Hybrid Piezoelectric-Pyroelectric Nanogenerator under Harsh Environments. ACS Appl. Mater. Interfaces.

[38] Lee S., Bae S.-H., Lin L., Ahn S., Park C., Kim S.-W., Cha S.N., Park Y.J., Wang Z.L. (2013). Flexible Hybrid Cell for Simultaneously Harvesting Thermal and Mechanical Energies. Nano Energy.

[39] Zhang Y., Shen S., Chiu C.Y., Murch R. (2019). Hybrid Rf-Solar Energy Harvesting Systems Utilizing Transparent Multiport Micromeshed Antennas. IEEE Trans. Microw. Theory Tech..

[40] Gu X., Hemour S., Wu K. Integrated Cooperative Radiofrequency (RF) and Kinetic Energy Harvester. Proceedings of the WPTC 2017—Wireless Power Transfer Conference.

[41] Wan S., Lu P., Yin D., Guan X., Ni W. (2023). A Compact Hybrid Solar and Electromagnetic Energy Harvester at 2.45 GHz Microstrip Rectenna. AEU—Int. J. Electron. Commun..

[42] Cambero E.V.V., Da Paz H.P., Da Silva V.S., De Araujo H.X., Casella I.R.S., Capovilla C.E. (2019). A 2.4 GHz Rectenna Based on a Solar Cell Antenna Array. IEEE Antennas Wirel. Propag. Lett..

[43] Niotaki K., Giuppi F., Georgiadis A., Collado A. (2014). Solar/EM Energy Harvester for Autonomous Operation of a Monitoring Sensor Platform. Wirel. Power Transf..

[44] Deo A., Reddy G.S., Arrawatia M., Shah P. A Partially Transparent GSM Band Loop RF-Solar Energy Harvester. Proceedings of the 2022 IEEE Microwaves, Antennas, and Propagation Conference (MAPCON).

[45] Yu B.Y., Wang Z.H., Ju L., Zhang C., Liu Z.G., Tao L., Lu W.B. (2022). Flexible and Wearable Hybrid RF and Solar Energy Harvesting System. IEEE Trans. Antennas Propag..

[46] Collado A., Georgiadis A. (2013). Conformal Hybrid Solar and Electromagnetic (EM) Energy Harvesting Rectenna. IEEE Trans. Circuits Syst. I Regul. Pap..

[47] Virili M., Georgiadis A., Collado A., Mezzanotte P., Roselli L. EM Characterization of a Patch Antenna with Thermo-Electric Generator and Solar Cell for Hybrid Energy Harvesting. Proceedings of the IEEE Radio and Wireless Symposium (RWS).

[48] Roy S., Tiang J.J., Roslee M.B., Ahmed M.T., Mahmud M.A.P. (2021). A Quad-Band Stacked Hybrid Ambient RF-Solar Energy Harvester with Higher RF-to-DC Rectification Efficiency. IEEE Access.

[49] Bito J., Bahr R., Hester J.G., Nauroze S.A., Georgiadis A., Tentzeris M.M. (2017). A Novel Solar and Electromagnetic Energy Harvesting System with a 3-D Printed Package for Energy Efficient Internet-of-Things Wireless Sensors. IEEE Trans. Microw. Theory Tech..

[50] Eteng A.A., Goh H.H., Rahim S.K.A., Alomainy A. (2021). A Review of Metasurfaces for Microwave Energy Transmission and Harvesting in Wireless Powered Networks. IEEE Access.

[51] Shi Y., Nan Y.H. (2022). Hybrid Power Harvesting From Ambient Radiofrequency and Solar Energy. IEEE Antennas Wirel. Propag. Lett..

[52] Meinzer N., Barnes W.L., Hooper I.R. (2014). Plasmonic Meta-Atoms and Metasurfaces. Nat. Photonics.

[53] Alavikia B., Almoneef T.S., Ramahi O.M. (2015). Complementary Split Ring Resonator Arrays for Electromagnetic Energy Harvesting. Appl. Phys. Lett..

[54] Zhong H.-T., Yang X.-X., Song X.-T., Guo Z.-Y., Yu F. (2017). Wideband Metamaterial Array with Polarization-Independent and Wide Incident Angle for Harvesting Ambient Electromagnetic Energy and Wireless Power Transfer. Appl. Phys. Lett..

[55] Ramahi O.M., Almoneef T.S., AlShareef M., Boybay M.S. (2012). Metamaterial Particles for Electromagnetic Energy Harvesting. Appl. Phys. Lett..

[56] Karaaslan M., Bağmancı M., Ünal E., Akgol O., Sabah C. (2017). Microwave Energy Harvesting Based on Metamaterial Absorbers with Multi-Layered Square Split Rings for Wireless Communications. Opt. Commun..

[57] Almoneef T.S., Ramahi O.M. Harvesting Electromagnetic Energy Using Metamaterial Particles. Proceedings of the IEEE Antennas and Propagation Society, AP-S International Symposium (Digest).

[58] Nowak M. (2021). Metamaterial-Based Sub-Microwave Electromagnetic Field Energy Harvesting System. Energies.

[59] Yoon G., Kim I., Rho J. (2016). Challenges in Fabrication towards Realization of Practical Metamaterials. Microelectron. Eng..

[60] Singh V., Khalily M., Tafazolli R. (2022). A Metasurface-Based Electronically Steerable Compact Antenna System with Reconfigurable Artificial Magnetic Conductor Reflector Elements. iScience.

[61] Viskadourakis Z., Tamiolakis E., Tsilipakos O., Tasolamprou A.C., Economou E.N., Kenanakis G. (2021). 3d-Printed Metasurface Units for Potential Energy Harvesting Applications at the 2.4 Ghz Frequency Band. Crystals.

[62] Fanourakis G., Markaki P., Theodosi A., Tsilipakos O., Viskadourakis Z., Kenanakis G. (2024). Engraved Complementary Toroidal Metasurfaces for Potential Energy Harvesting Applications in Microwave Band. J. Appl. Phys..

[63] Ishikawa A., Kato T., Takeyasu N., Fujimori K., Tsuruta K. (2017). Selective Electroless Plating of 3D-Printed Plastic Structures for Three-Dimensional Microwave Metamaterials. Appl. Phys. Lett..

[64] Zhang K.P., Liao Y.F., Qiu B., Zheng Y.K., Yu L.K., He G.H., Chen Q.N., Sun D.H. (2021). 3D Printed Embedded Metamaterials. Small.

[65] Oumbé Tékam G.T., Ginis V., Danckaert J., Tassin P. (2017). Designing an Efficient Rectifying Cut-Wire Metasurface for Electromagnetic Energy Harvesting. Appl. Phys. Lett..

[66] Pandey R., Shankhwar A.K., Singh A. (2021). An Improved Conversion Efficiency of 1.975 to 4.744 GHz Rectenna for Wireless Sensor Applications. Prog. Electromagn. Res. C.

[67] Zhang B., Jiang W., Yu C., Liu C. (2015). A C-Band Microwave Rectifier without Capacitors for Microwave Power Transmission. Int. J. Microw. Wirel. Technol..

[68] Duan X., Chen X., Zhou L. (2016). A Metamaterial Electromagnetic Energy Rectifying Surface with High Harvesting Efficiency. AIP Adv..

[69] Virili M., Georgiadis A., Mira F., Collado A., Alimenti F., Mezzanotte P., Roselli L. EH Performance of an Hybrid Energy Harvester for Autonomous Nodes. Proceedings of the 2016 IEEE Topical Conference on Wireless Sensors and Sensor Networks (WiSNet).

[70] Duran E., Piliougine M., Sidrach-de-Cardona M., Galan J., Andujar J.M. Different Methods to Obtain the I–V Curve of PV Modules: A Review. Proceedings of the 2008 33rd IEEE Photovoltaic Specialists Conference.

[71] Mujahidin I., Kitagawa A. (2021). The Novel CPW 2.4 GHz Antenna with Parallel Hybrid Electromagnetic Solar for IoT Energy Harvesting and Wireless Sensors. Int. J. Adv. Comput. Sci. Appl..

[72] Naresh B., Singh V.K., Sharma V.K. (2021). Integration of RF Rectenna with Thin Film Solar Cell to Power Wearable Electronics. Int. J. Microw. Wirel. Technol..

[73] Hirata H., Walczak T., Swartz H.M. (2000). Electronically Tunable Surface-Coil-Type Resonator for L-Band EPR Spectroscopy. J. Magn. Reson..

[74] Zhang P., Zhang X., Li L. (2022). An Optically Transparent Metantenna for RF Wireless Energy Harvesting. IEEE Trans. Antennas Propag..

[75] Al-Adhami Y., Erçelebi E. (2017). Plasmonic Metamaterial Dipole Antenna Array Circuitry Based on Flexible Solar Cell Panel for Self-powered Wireless Systems. Microw. Opt. Technol. Lett..

[76] Shen S., Zhang Y., Chiu C.-Y., Murch R. (2019). A Triple-Band High-Gain Multibeam Ambient RF Energy Harvesting System Utilizing Hybrid Combining. IEEE Trans. Ind. Electron..

[77] He Z., Lan J., Liu C. (2021). Compact Rectifiers with Ultra-Wide Input Power Range Based on Nonlinear Impedance Characteristics of Schottky Diodes. IEEE Trans. Power Electron..

[78] Qi M.Q., Tang W.X., Ma H.F., Pan B.C., Tao Z., Sun Y.Z., Cui T.J. (2015). Suppressing Side-Lobe Radiations of Horn Antenna by Loading Metamaterial Lens. Sci. Rep..

[79] Al-Adhami Y., Erçelebi E. (2018). A Plasmonic Monopole Antenna Array on Flexible Photovoltaic Panels for Further Use of the Green Energy Harvesting. Prog. Electromagn. Res. M.

[80] Li K., Bi M., Tian H., Liu Y., Zhu H., Jiang T. (2024). A Hybrid Energy Supply System Based on Metamaterial Antenna Integrated Solar Cells for IoT Nodes. Sustain. Energy Technol. Assess..

[81] Hameed Z., Moez K. (2017). Design of Impedance Matching Circuits for RF Energy Harvesting Systems. Microelectron. J..

[82] Veloo S.G., Tiang J.J., Muhammad S., Wong S.K. (2023). A Hybrid Solar-RF Energy Harvesting System Based on an EM4325-Embedded RFID Tag. Electronics.

[83] Viskadourakis Z., Grammatikakis K., Katsara K., Drymiskianaki A., Kenanakis G. (2022). Fabrication of Metasurfaces on Building Construction Materials for Potential Electromagnetic Applications in the Microwave Band. Materials.

[84] Bošnjaković M., Stojkov M., Katinić M., Lacković I. (2023). Effects of Extreme Weather Conditions on PV Systems. Sustainability.

[85] Jordan D.C., Perry K., White R., Deline C. (2023). Extreme Weather and PV Performance. IEEE J. Photovolt..

[86] Gholami A., Ameri M., Zandi M., Ghoachani R.G., Gerashi S.J., Kazem H.A., Al-Waeli A.H.A. (2023). Impact of Harsh Weather Conditions on Solar Photovoltaic Cell Temperature: Experimental Analysis and Thermal-Optical Modeling. Sol. Energy.

[87] Su L., Vélez P., Casacuberta P., Muñoz-Enano J., Martín F. (2023). Microwave Humidity Sensor for Early Detection of Sweat and Urine Leakage. Electronics.

[88] Park J.-K., Kang T.-G., Kim B.-H., Lee H.-J., Choi H.H., Yook J.-G. (2018). Real-Time Humidity Sensor Based on Microwave Resonator Coupled with PEDOT: PSS Conducting Polymer Film. Sci. Rep..

[89] Keshavarz R., Lipman J., Schreurs D.M.-P., Shariati N. (2021). Highly Sensitive Differential Microwave Sensor for Soil Moisture Measurement. IEEE Sens. J..

