# Supplementary material for: Hybrid Microwave/Solar Energy Harvesting System Using 3D-Printed Metasurfaces
Source: Materials (Basel). 2024 Dec 5;17(23):5969. doi: 10.3390/ma17235969 (PMC11643723; doi:10.3390/ma17235969)
Supplement: Supplementary file 1 [file materials-17-05969-s001.zip › materials-3305861-supplementary.pdf]

Hybrid microwave / Solar energy harvesting system using 3D - printed metamaterials

Argyri Drymiskianaki[1, 2, *], Zacharias Viskadourakis[2] and George Kenanakis[2,*]

[1] Department of Materials Science and Technology, University of Crete, GR-70013 Heraklion, Crete, Greece
[2] Institute of Electronic Structure and Laser (IESL)—Foundation for Research and Technology—Hellas (FORTH), 100 N. Plastira, Vassilika Vouton, GR-70013 Heraklion, Crete, Greece
* Correspondence: adrym@materials.uoc.gr (A.D.); gkenanak@iesl.forth.gr (G.K.)

Supplementary material

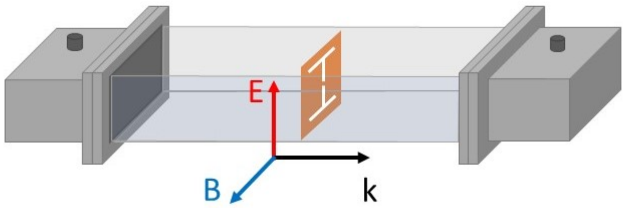

Figure S1: Orientation of the meta-atom into the waveguide, during its electromagnetic behavior study

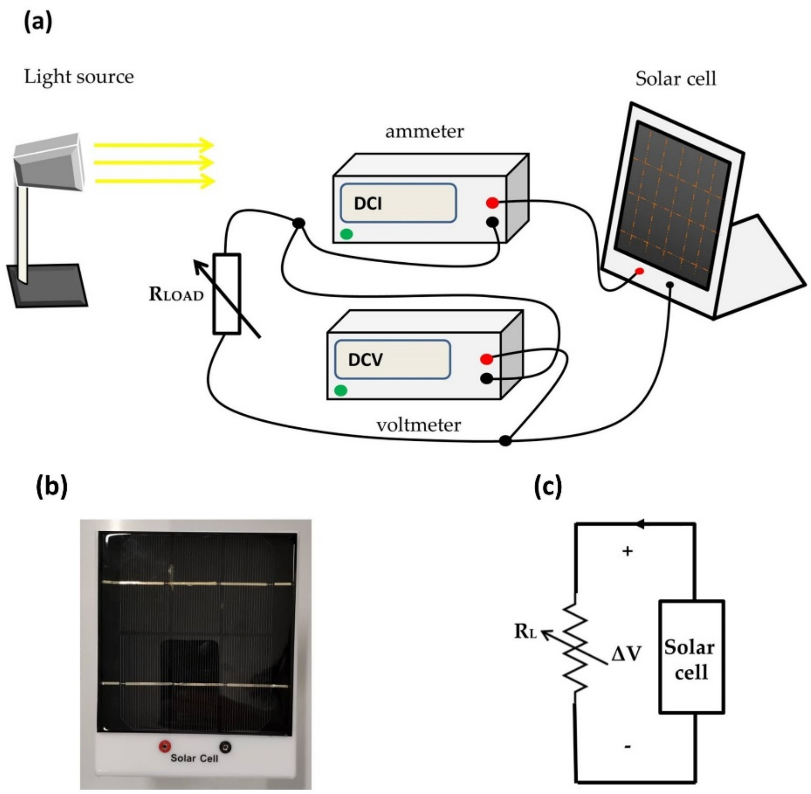

Figure S2. (a) Schematic representation of the experimental setup used to derive the I-V characteristics of the solar cell. (b) The amorphous Si solar cell used in the HEH system. (c) The equivalent circuit diagram of Figure S2a.

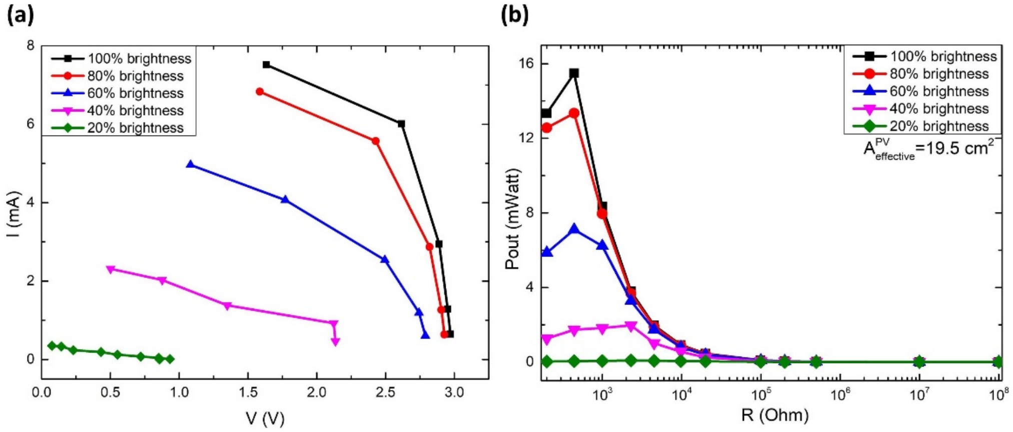

Figure S3. (a) I-V characteristics of the PV module at different lighting conditions. The effective area of the solar cell was reduced to match the affective area of the MS. (b) Output power over resistance load at various brightness levels

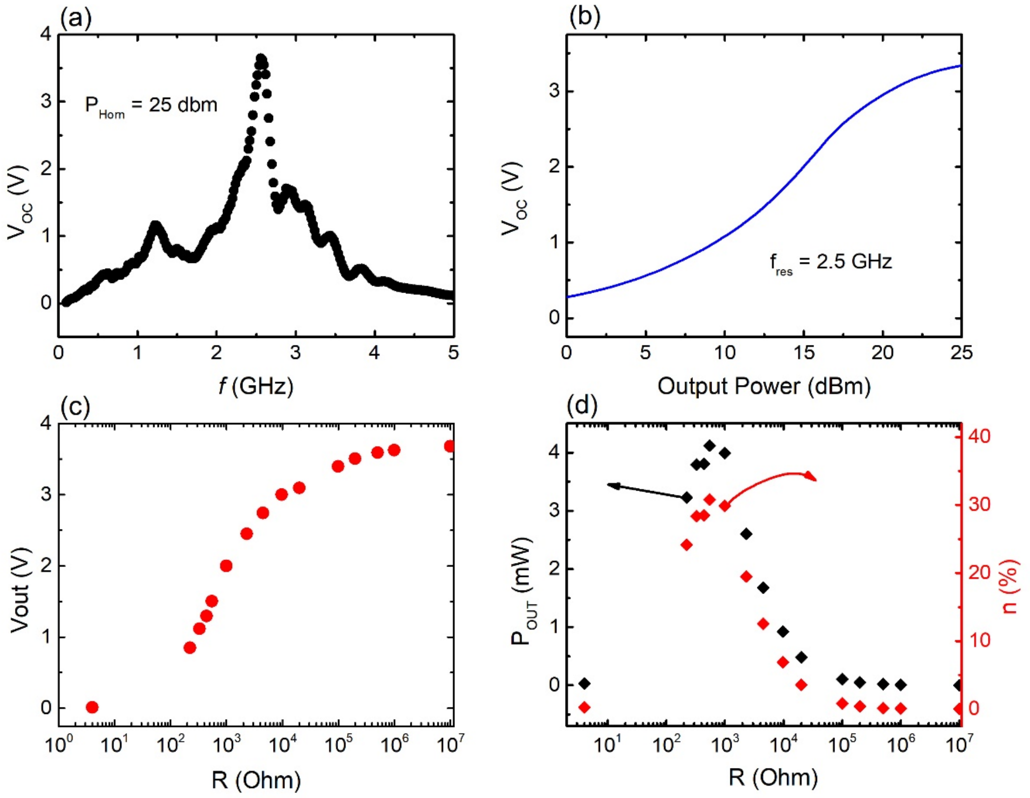

Figure S4. (a) Open-circuit voltage (V_{OC}) vs. frequency for PCB-grown, single metasurface unit. (b) V_{OC} as a function of the horn antenna output power. (c) The dependence of output voltage (V_{OUT}) on the resistance load, at resonance frequency. (d) Output power (black points) and calculated efficiency (red points) as a function of resistance load for the single metasurface unit.
